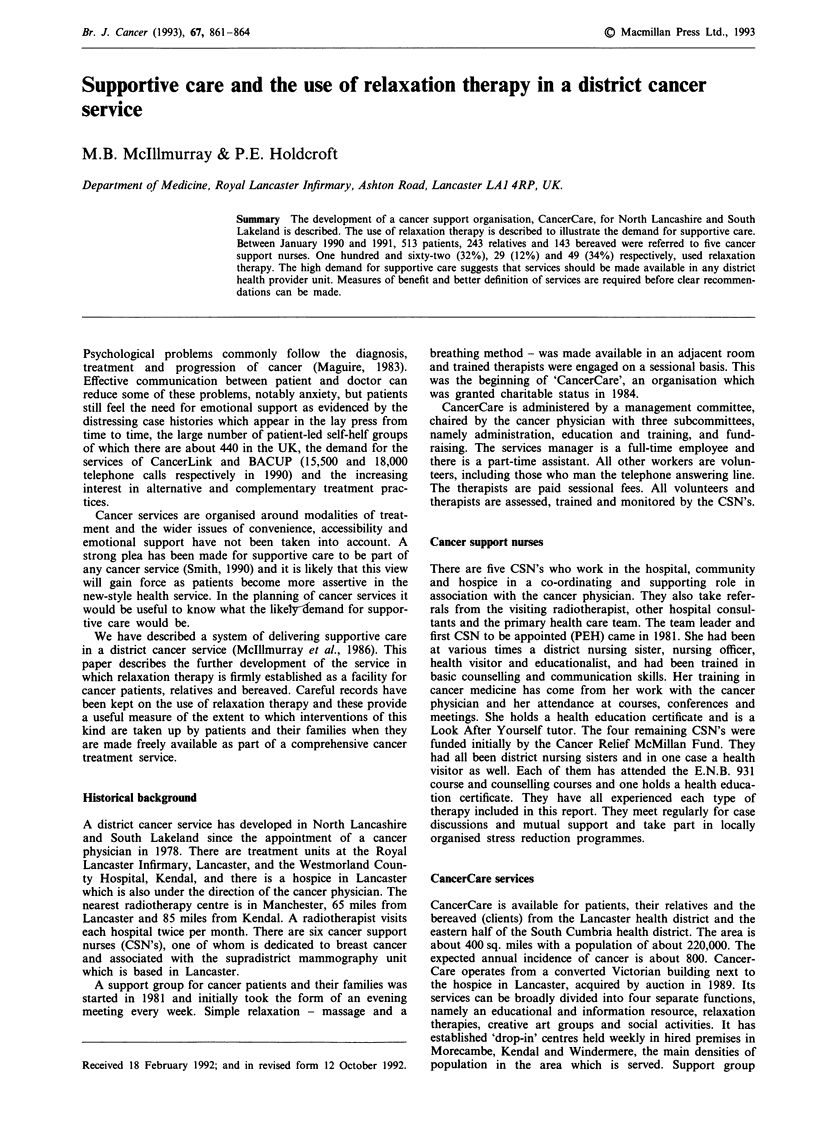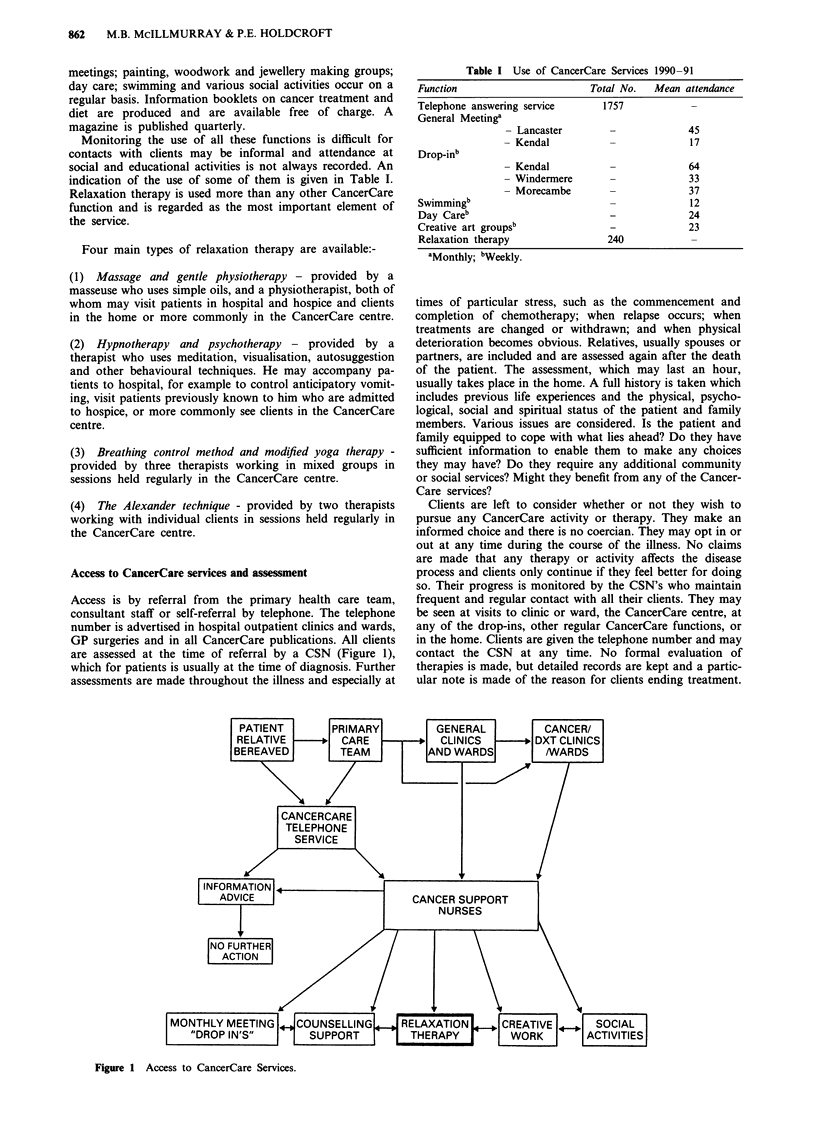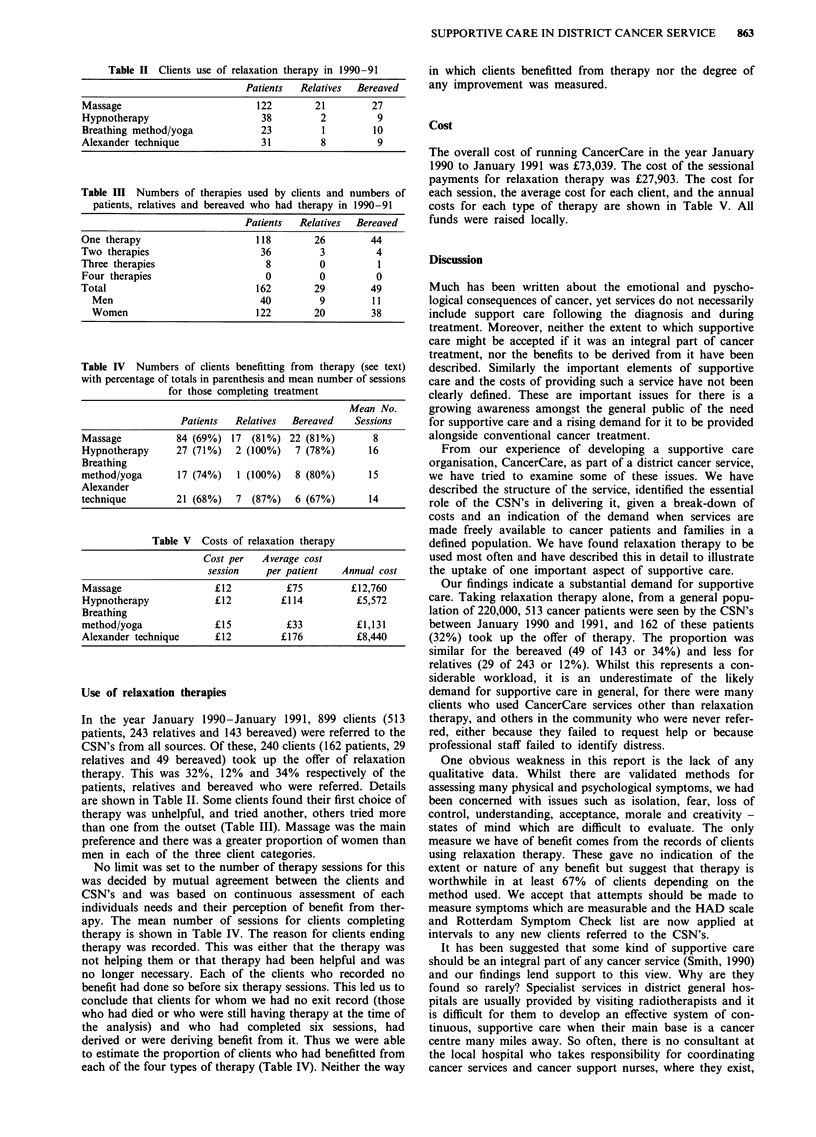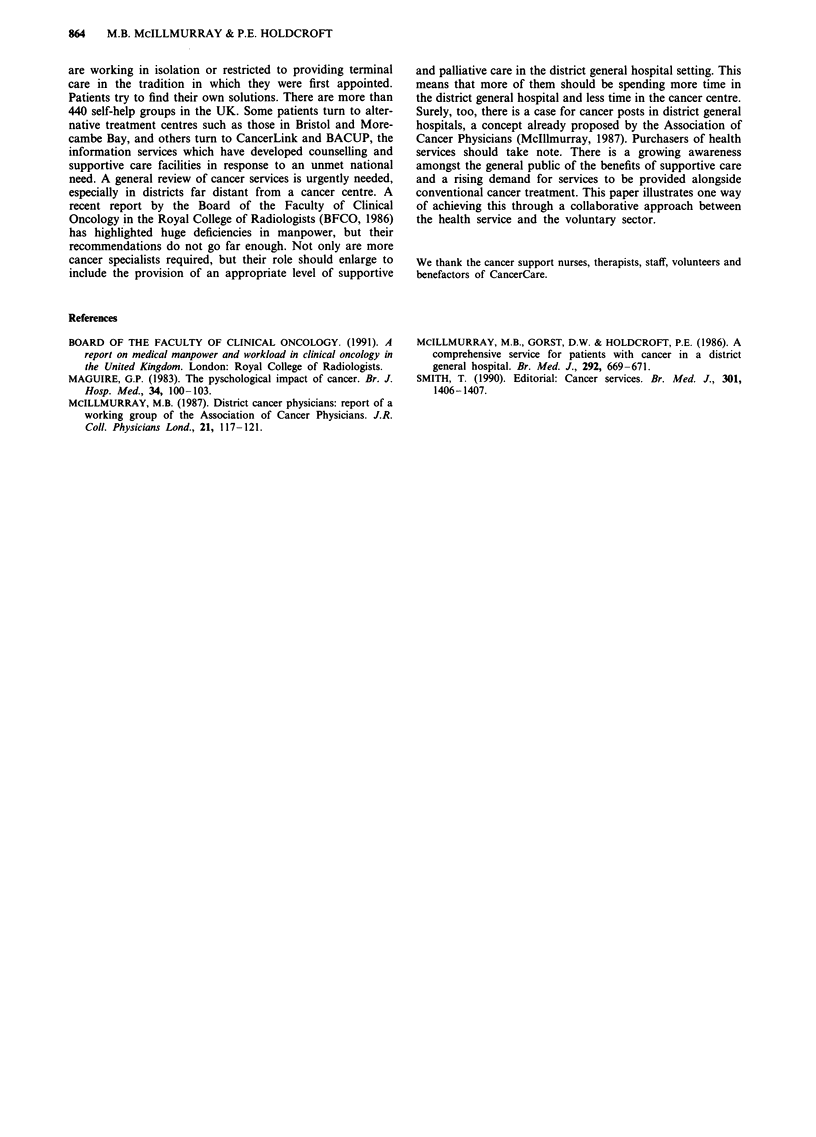# Supportive care and the use of relaxation therapy in a district cancer service.

**DOI:** 10.1038/bjc.1993.158

**Published:** 1993-04

**Authors:** M. B. McIllmurray, P. E. Holdcroft

**Affiliations:** Department of Medicine, Royal Lancaster Infirmary, UK.

## Abstract

The development of a cancer support organisation, CancerCare, for North Lancashire and South Lakeland is described. The use of relaxation therapy is described to illustrate the demand for supportive care. Between January 1990 and 1991, 513 patients, 243 relatives and 143 bereaved were referred to five cancer support nurses. One hundred and sixty-two (32%), 29 (12%) and 49 (34%) respectively, used relaxation therapy. The high demand for supportive care suggests that services should be made available in any district health provider unit. Measures of benefit and better definition of services are required before clear recommendations can be made.


© Macmillan Press Ltd., 1993

### Supportive care and the use of relaxation therapy in a district cancer service

M.B. McIllmurray & P.E. Holdcroft

*Department of Medicine, Royal Lancaster Infirmary, Ashton Road, Lancaster LA1 4RP, UK.*

**Summary** The development of a cancer support organisation, CancerCare, for North Lancashire and South Lakeland is described. The use of relaxation therapy is described to illustrate the demand for supportive care. Between January 1990 and 1991, 513 patients, 243 relatives and 143 bereaved were referred to five cancer support nurses. One hundred and sixty-two (32%), 29 (12%) and 49 (34%) respectively, used relaxation therapy. The high demand for supportive care suggests that services should be made available in any district health provider unit. Measures of benefit and better definition of services are required before clear recommendations can be made.

Psychological problems commonly follow the diagnosis, treatment and progression of cancer (Maguire, 1983). Effective communication between patient and doctor can reduce some of these problems, notably anxiety, but patients still feel the need for emotional support as evidenced by the distressing case histories which appear in the lay press from time to time, the large number of patient-led self-helf groups of which there are about 440 in the UK, the demand for the services of CancerLink and BACUP (15,500 and 18,000 telephone calls respectively in 1990) and the increasing interest in alternative and complementary treatment practices.

Cancer services are organised around modalities of treatment and the wider issues of convenience, accessibility and emotional support have not been taken into account. A strong plea has been made for supportive care to be part of any cancer service (Smith, 1990) and it is likely that this view will gain force as patients become more assertive in the new-style health service. In the planning of cancer services it would be useful to know what the likely demand for supportive care would be.

We have described a system of delivering supportive care in a district cancer service (McIllmurray *et al.*, 1986). This paper describes the further development of the service in which relaxation therapy is firmly established as a facility for cancer patients, relatives and bereaved. Careful records have been kept on the use of relaxation therapy and these provide a useful measure of the extent to which interventions of this kind are taken up by patients and their families when they are made freely available as part of a comprehensive cancer treatment service.

### Historical background

A district cancer service has developed in North Lancashire and South Lakeland since the appointment of a cancer physician in 1978. There are treatment units at the Royal Lancaster Infirmary, Lancaster, and the Westmorland County Hospital, Kendal, and there is a hospice in Lancaster which is also under the direction of the cancer physician. The nearest radiotherapy centre is in Manchester, 65 miles from Lancaster and 85 miles from Kendal. A radiotherapist visits each hospital twice per month. There are six cancer support nurses (CSN's), one of whom is dedicated to breast cancer and associated with the supradistrict mammography unit which is based in Lancaster.

A support group for cancer patients and their families was started in 1981 and initially took the form of an evening meeting every week. Simple relaxation – massage and a breathing method – was made available in an adjacent room and trained therapists were engaged on a sessional basis. This was the beginning of 'CancerCare', an organisation which was granted charitable status in 1984.

CancerCare is administered by a management committee, chaired by the cancer physician with three subcommittees, namely administration, education and training, and fund-raising. The services manager is a full-time employee and there is a part-time assistant. All other workers are volunteers, including those who man the telephone answering line. The therapists are paid sessional fees. All volunteers and therapists are assessed, trained and monitored by the CSN's.

### Cancer support nurses

There are five CSN's who work in the hospital, community and hospice in a co-ordinating and supporting role in association with the cancer physician. They also take referrals from the visiting radiotherapist, other hospital consultants and the primary health care team. The team leader and first CSN to be appointed (PEH) came in 1981. She had been at various times a district nursing sister, nursing officer, health visitor and educationalist, and had been trained in basic counselling and communication skills. Her training in cancer medicine has come from her work with the cancer physician and her attendance at courses, conferences and meetings. She holds a health education certificate and is a Look After Yourself tutor. The four remaining CSN's were funded initially by the Cancer Relief McMillan Fund. They had all been district nursing sisters and in one case a health visitor as well. Each of them has attended the E.N.B. 931 course and counselling courses and one holds a health education certificate. They have all experienced each type of therapy included in this report. They meet regularly for case discussions and mutual support and take part in locally organised stress reduction programmes.

### CancerCare services

CancerCare is available for patients, their relatives and the bereaved (clients) from the Lancaster health district and the eastern half of the South Cumbria health district. The area is about 400 sq. miles with a population of about 220,000. The expected annual incidence of cancer is about 800. CancerCare operates from a converted Victorian building next to the hospice in Lancaster, acquired by auction in 1989. Its services can be broadly divided into four separate functions, namely an educational and information resource, relaxation therapies, creative art groups and social activities. It has established 'drop-in' centres held weekly in hired premises in Morecambe, Kendal and Windermere, the main densities of population in the area which is served. Support group

Received 18 February 1992; and in revised form 12 October 1992.

meetings; painting, woodwork and jewellery making groups; day care; swimming and various social activities occur on a regular basis. Information booklets on cancer treatment and diet are produced and are available free of charge. A magazine is published quarterly.

Monitoring the use of all these functions is difficult for contacts with clients may be informal and attendance at social and educational activities is not always recorded. An indication of the use of some of them is given in Table I. Relaxation therapy is used more than any other CancerCare function and is regarded as the most important element of the service.

Four main types of relaxation therapy are available:-

(1) *Massage and gentle physiotherapy* – provided by a masseuse who uses simple oils, and a physiotherapist, both of whom may visit patients in hospital and hospice and clients in the home or more commonly in the CancerCare centre.

(2) *Hypnotherapy and psychotherapy* – provided by a therapist who uses meditation, visualisation, autosuggestion and other behavioural techniques. He may accompany patients to hospital, for example to control anticipatory vomiting, visit patients previously known to him who are admitted to hospice, or more commonly see clients in the CancerCare centre.

(3) *Breathing control method and modified yoga therapy* - provided by three therapists working in mixed groups in sessions held regularly in the CancerCare centre.

(4) *The Alexander technique* - provided by two therapists working with individual clients in sessions held regularly in the CancerCare centre.

**Table I** Use of CancerCare Services 1990–91

| *Function* | *Total No.* | *Mean attendance* |
|---|---|---|
| Telephone answering service | 1757 | – |
| General Meeting[a] | | |
| – Lancaster | – | 45 |
| – Kendal | – | 17 |
| Drop-in[b] | | |
| – Kendal | – | 64 |
| – Windermere | – | 33 |
| – Morecambe | – | 37 |
| Swimming[b] | – | 12 |
| Day Care[b] | – | 24 |
| Creative art groups[b] | – | 23 |
| Relaxation therapy | 240 | – |

[a]Monthly; [b]Weekly.

### Access to CancerCare services and assessment

Access is by referral from the primary health care team, consultant staff or self-referral by telephone. The telephone number is advertised in hospital outpatient clinics and wards, GP surgeries and in all CancerCare publications. All clients are assessed at the time of referral by a CSN (Figure 1), which for patients is usually at the time of diagnosis. Further assessments are made throughout the illness and especially at times of particular stress, such as the commencement and completion of chemotherapy; when relapse occurs; when treatments are changed or withdrawn; and when physical deterioration becomes obvious. Relatives, usually spouses or partners, are included and are assessed again after the death of the patient. The assessment, which may last an hour, usually takes place in the home. A full history is taken which includes previous life experiences and the physical, psychological, social and spiritual status of the patient and family members. Various issues are considered. Is the patient and family equipped to cope with what lies ahead? Do they have sufficient information to enable them to make any choices they may have? Do they require any additional community or social services? Might they benefit from any of the CancerCare services?

Clients are left to consider whether or not they wish to pursue any CancerCare activity or therapy. They make an informed choice and there is no coercian. They may opt in or out at any time during the course of the illness. No claims are made that any therapy or activity affects the disease process and clients only continue if they feel better for doing so. Their progress is monitored by the CSN's who maintain frequent and regular contact with all their clients. They may be seen at visits to clinic or ward, the CancerCare centre, at any of the drop-ins, other regular CancerCare functions, or in the home. Clients are given the telephone number and may contact the CSN at any time. No formal evaluation of therapies is made, but detailed records are kept and a particular note is made of the reason for clients ending treatment.

**Figure 1** Access to CancerCare Services.

**Table II** Clients use of relaxation therapy in 1990–91

| | *Patients* | *Relatives* | *Bereaved* |
|---|---|---|---|
| Massage | 122 | 21 | 27 |
| Hypnotherapy | 38 | 2 | 9 |
| Breathing method/yoga | 23 | 1 | 10 |
| Alexander technique | 31 | 8 | 9 |

**Table III** Numbers of therapies used by clients and numbers of patients, relatives and bereaved who had therapy in 1990–91

| | *Patients* | *Relatives* | *Bereaved* |
|---|---|---|---|
| One therapy | 118 | 26 | 44 |
| Two therapies | 36 | 3 | 4 |
| Three therapies | 8 | 0 | 1 |
| Four therapies | 0 | 0 | 0 |
| Total | 162 | 29 | 49 |
| Men | 40 | 9 | 11 |
| Women | 122 | 20 | 38 |

**Table IV** Numbers of clients benefitting from therapy (see text) with percentage of totals in parenthesis and mean number of sessions for those completing treatment

| | *Patients* | *Relatives* | *Bereaved* | *Mean No. Sessions* |
|---|---|---|---|---|
| Massage | 84 (69%) | 17 (81%) | 22 (81%) | 8 |
| Hypnotherapy | 27 (71%) | 2 (100%) | 7 (78%) | 16 |
| Breathing method/yoga | 17 (74%) | 1 (100%) | 8 (80%) | 15 |
| Alexander technique | 21 (68%) | 7 (87%) | 6 (67%) | 14 |

**Table V** Costs of relaxation therapy

| | *Cost per session* | *Average cost per patient* | *Annual cost* |
|---|---|---|---|
| Massage | £12 | £75 | £12,760 |
| Hypnotherapy | £12 | £114 | £5,572 |
| Breathing method/yoga | £15 | £33 | £1,131 |
| Alexander technique | £12 | £176 | £8,440 |

### Use of relaxation therapies

In the year January 1990–January 1991, 899 clients (513 patients, 243 relatives and 143 bereaved) were referred to the CSN's from all sources. Of these, 240 clients (162 patients, 29 relatives and 49 bereaved) took up the offer of relaxation therapy. This was 32%, 12% and 34% respectively of the patients, relatives and bereaved who were referred. Details are shown in Table II. Some clients found their first choice of therapy was unhelpful, and tried another, others tried more than one from the outset (Table III). Massage was the main preference and there was a greater proportion of women than men in each of the three client categories.

No limit was set to the number of therapy sessions for this was decided by mutual agreement between the clients and CSN's and was based on continuous assessment of each individuals needs and their perception of benefit from therapy. The mean number of sessions for clients completing therapy is shown in Table IV. The reason for clients ending therapy was recorded. This was either that the therapy was not helping them or that therapy had been helpful and was no longer necessary. Each of the clients who recorded no benefit had done so before six therapy sessions. This led us to conclude that clients for whom we had no exit record (those who had died or who were still having therapy at the time of the analysis) and who had completed six sessions, had derived or were deriving benefit from it. Thus we were able to estimate the proportion of clients who had benefitted from each of the four types of therapy (Table IV). Neither the way in which clients benefitted from therapy nor the degree of any improvement was measured.

### Cost

The overall cost of running CancerCare in the year January 1990 to January 1991 was £73,039. The cost of the sessional payments for relaxation therapy was £27,903. The cost for each session, the average cost for each client, and the annual costs for each type of therapy are shown in Table V. All funds were raised locally.

### Discussion

Much has been written about the emotional and pyschological consequences of cancer, yet services do not necessarily include support care following the diagnosis and during treatment. Moreover, neither the extent to which supportive care might be accepted if it was an integral part of cancer treatment, nor the benefits to be derived from it have been described. Similarly the important elements of supportive care and the costs of providing such a service have not been clearly defined. These are important issues for there is a growing awareness amongst the general public of the need for supportive care and a rising demand for it to be provided alongside conventional cancer treatment.

From our experience of developing a supportive care organisation, CancerCare, as part of a district cancer service, we have tried to examine some of these issues. We have described the structure of the service, identified the essential role of the CSN's in delivering it, given a break-down of costs and an indication of the demand when services are made freely available to cancer patients and families in a defined population. We have found relaxation therapy to be used most often and have described this in detail to illustrate the uptake of one important aspect of supportive care.

Our findings indicate a substantial demand for supportive care. Taking relaxation therapy alone, from a general population of 220,000, 513 cancer patients were seen by the CSN's between January 1990 and 1991, and 162 of these patients (32%) took up the offer of therapy. The proportion was similar for the bereaved (49 of 143 or 34%) and less for relatives (29 of 243 or 12%). Whilst this represents a considerable workload, it is an underestimate of the likely demand for supportive care in general, for there were many clients who used CancerCare services other than relaxation therapy, and others in the community who were never referred, either because they failed to request help or because professional staff failed to identify distress.

One obvious weakness in this report is the lack of any qualitative data. Whilst there are validated methods for assessing many physical and psychological symptoms, we had been concerned with issues such as isolation, fear, loss of control, understanding, acceptance, morale and creativity – states of mind which are difficult to evaluate. The only measure we have of benefit comes from the records of clients using relaxation therapy. These gave no indication of the extent or nature of any benefit but suggest that therapy is worthwhile in at least 67% of clients depending on the method used. We accept that attempts should be made to measure symptoms which are measurable and the HAD scale and Rotterdam Symptom Check list are now applied at intervals to any new clients referred to the CSN's.

It has been suggested that some kind of supportive care should be an integral part of any cancer service (Smith, 1990) and our findings lend support to this view. Why are they found so rarely? Specialist services in district general hospitals are usually provided by visiting radiotherapists and it is difficult for them to develop an effective system of continuous, supportive care when their main base is a cancer centre many miles away. So often, there is no consultant at the local hospital who takes responsibility for coordinating cancer services and cancer support nurses, where they exist, are working in isolation or restricted to providing terminal care in the tradition in which they were first appointed. Patients try to find their own solutions. There are more than 440 self-help groups in the UK. Some patients turn to alternative treatment centres such as those in Bristol and Morecambe Bay, and others turn to CancerLink and BACUP, the information services which have developed counselling and supportive care facilities in response to an unmet national need. A general review of cancer services is urgently needed, especially in districts far distant from a cancer centre. A recent report by the Board of the Faculty of Clinical Oncology in the Royal College of Radiologists (BFCO, 1986) has highlighted huge deficiencies in manpower, but their recommendations do not go far enough. Not only are more cancer specialists required, but their role should enlarge to include the provision of an appropriate level of supportive and palliative care in the district general hospital setting. This means that more of them should be spending more time in the district general hospital and less time in the cancer centre. Surely, too, there is a case for cancer posts in district general hospitals, a concept already proposed by the Association of Cancer Physicians (McIllmurray, 1987). Purchasers of health services should take note. There is a growing awareness amongst the general public of the benefits of supportive care and a rising demand for services to be provided alongside conventional cancer treatment. This paper illustrates one way of achieving this through a collaborative approach between the health service and the voluntary sector.

We thank the cancer support nurses, therapists, staff, volunteers and benefactors of CancerCare.

### References

BOARD OF THE FACULTY OF CLINICAL ONCOLOGY. (1991). *A report on medical manpower and workload in clinical oncology in the United Kingdom*. London: Royal College of Radiologists.

MAGUIRE, G.P. (1983). The pyschological impact of cancer. *Br. J. Hosp. Med.*, **34**, 100–103.

McILLMURRAY, M.B. (1987). District cancer physicians: report of a working group of the Association of Cancer Physicians. *J.R. Coll. Physicians Lond.*, **21**, 117–121.

McILLMURRAY, M.B., GORST, D.W. & HOLDCROFT, P.E. (1986). A comprehensive service for patients with cancer in a district general hospital. *Br. Med. J.*, **292**, 669–671.

SMITH, T. (1990). Editorial: Cancer services. *Br. Med. J.*, **301**, 1406–1407.